# Data from SILAC-based quantitative analysis of lysates from mouse microglial cells treated with Withaferin A (WA)

**DOI:** 10.1016/j.dib.2016.03.026

**Published:** 2016-03-14

**Authors:** Malathi Narayan, Kent W. Seeley, Umesh K. Jinwal

**Affiliations:** a Department of Pharmaceutical Sciences, College of Pharmacy, Byrd Alzheimer׳s Institute, University of South Florida-Health, 4001 E. Fletcher Ave, MDC36, Tampa, FL 33613, USA; b Florida Center of Excellence for Drug Discovery & Innovation at the University of South Florida, 3720 Spectrum Blvd., Suite 303, IDR Building, Tampa, FL 33612, USA

**Keywords:** Withaferin A, Microglial cell line, SILAC, Proteomics, Mass spectrometry

## Abstract

Mass spectrometry data collected in a study analyzing the effect of withaferin A (WA) on a mouse microglial (N9) cell line is presented in this article. Data was collected from SILAC-based quantitative analysis of lysates from mouse microglial cells treated with either WA or DMSO vehicle control. This article reports all the proteins that were identified in this analysis. The data presented here is related to the published research article on the effect of WA on the differential regulation of proteins in mouse microglial cells [1]. Mass spectrometry data has also been deposited in the ProteomeXchange with the identifier PXD003032.

**Specifications Table**

**Table:** 

Subject area	*Biology*
More specific subject area	*Proteomics*
Type of data	*Mass spectrometry*
How data was acquired	*MS data was acquired on a LTQ Orbitrap XL (Thermo) mass spectrometer*
Data format	Raw files (*raw) and Excel files (.xlsx)
Experimental factors	*N9 mouse microglial cells were grown in either light (*^*12*^*C*_*6*_*l**-lysine and*^*12*^*C*_*6*_^*14*^*N*_*4*_*l-arginine) or heavy (*^*13*^*C*_*6*_*l**-lysine and*^*12*^*C*_*6*_^*14*^*N*_*4*_*l-arginine) media. Light and heavy labeled cells were treated either with DMSO or WA for 24 hours, respectively.*
Experimental features	*After treatment, cells were lysed and the lysates were digested with trypsin using the filter-aided sample preparation (FASP) kit. Peptides were fractionated using a strong cation-exchange (SCX) column and analyzed by LC–MS/MS.*
Data source location	*Tampa, Florida, USA*
Data accessibility	*Data in this article has been deposited in the ProteomeXchange with the identifier ProteomeXchange: PXD003032.*

**Value of the data**

• Analysis of the global effect of WA on the proteome of a microglial cell line.
• Quantitative analysis of these data shows unique signaling pathways initiated by WA.
• Data provide a baseline for the effect of WA on resting microglia, which might be useful for other investigator to compare with the effect of WA on activated microglia in Alzheimer׳s, Parkinson׳s and several other related diseases.

## 1 Data

**Fig. 1:**
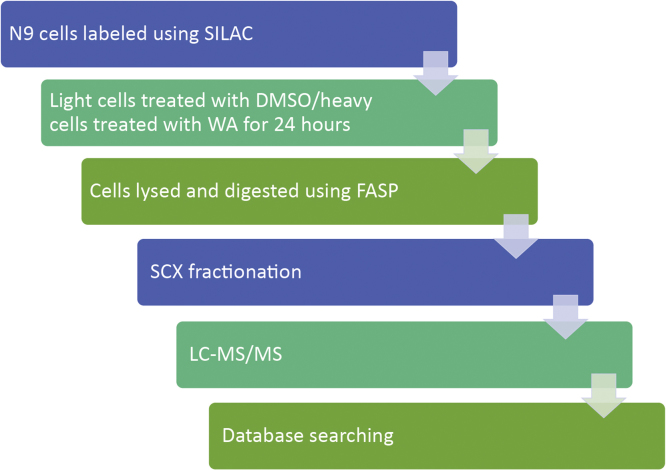
A flowchart describing the experimental design for the analysis of the effect of WA on N9 cells.

This paper reports mass spectrometry data on the differential regulation of proteins induced by the treatment of N9 mouse microglial cells with WA. Data were generated using SILAC-based approach and analyzed as described below and outlined in Fig. 1.

## 2 Experimental design, materials and methods

We labeled cells with either light or heavy SILAC media. The light labeled cells were treated with DMSO vehicle control and the heavy labeled cells were treated with 2 WA for 24 h. Cells were then lysed, and the light and heavy lysates were combined and digested with trypsin. The resulting peptides were fractionated and analyzed by LC–MS/MS. The raw mass spectrometry data was then analyzed using MaxQuant. The experimental design in outlined in Fig. 1.

### 2.1 SILAC labeling of N9 mouse microglial cells

Culturing conditions and maintenance of N9 cells are described in detail [1]. Briefly, cells were cultured in DMEM containing 10% FBS, l-glutamine, and penicillin and streptomycin. For stable isotope labeling of amino acids in cell culture (SILAC) with heavy and light amino acids [2] the Pierce SILAC Protein Quantitation Kit – DMEM (Thermo Scientific, Waltham, MA) was used as per manufacturer׳s instructions. Cells were grown in DMEM containing 10% dialyzed fetal bovine serum (FBS), penicillin/streptomycin, ^12^C_6_ l-lysine and ^12^C_6_^14^N_4_ l-arginine for light labeling or ^13^C_6_ l-lysine and ^12^C_6_^14^N_4_ l-arginine for heavy labeling for at least five cell doublings for efficient incorporation of the label. Mass spectrometry analysis was used to determine the efficiency of labeling to be >98%.

As mentioned earlier [1], WA (2 µM) or DMSO vehicle control treatments were performed in triplicate for 24 hours on light and heavy labeled cells, respectively. After trypsin treatment cell pellets were lysed in a lysis buffer consisting of Component 1:125 mM Tris–HCl, pH 7.6, and Component 2: 20% SDS+500 mM dithiothreitol (DTT, which needs to be added fresh). Components 1 and 2 were added to the cell pellet in a ratio of 80:20 (i.e. 80 µl Component 1:20 µl Component 2) resulting a final concentration of 100 mM Tris–HCl, 4% SDS and 100 mM DTT. Samples were heated at 100 °C for 5 minutes. For complete lysis sonication performed in 30 second intervals for six times at 20% amplitude with a one minute incubation on ice in between pulses. Samples were centrifuged for 20 min at 15,000×*g* at 4 °C. The supernatant was used for protein estimation. Due to presence of SDS in the lysis buffer, the ionic detergent compatibility reagent (Thermo Scientific) was used with the Pierce 660 nm assay for protein estimation.

### 2.2 Sample preparation using filter-aided sample preparation and desalting

Filter-aided sample preparation (FASP; Protein Discovery, Knoxville TN), as described by Wisniewski and Mann [3], was used to digest whole cell lysates. A total of 400 µg the protein sample in a 30 µl volume was mixed with 200 µL 8 M urea and added to the 30 kDa FASP spin filter for buffer exchange. Samples were alkylated with iodoacetamide for 30 min in the dark and buffer exchanged with 3×100 µL additions of 50 mM ammonium bicarbonate by centrifugation at 14 000×*g* for 10 min. Trypsin was added at 1:100 and samples were incubated overnight at 37 °C. Peptides were collected by centrifugation by addition of 2×40 µL 50 mM ammonium bicarbonate and 40 µL NaCl. The peptide mixture was acidified by addition of formic acid to pH 3. Desalted was performed using Supelco Discovery DSC-18 SPE (Supelco, Bellefonte, PA) columns in combination with a Supelco vacuum manifold. Using vacuum concentrator (Thermo) peptides were dried and then resuspended in 20 µL of formic acid solution (0.1% formic acid in H_2_O). A Thermo Surveyor HPLC system with a 15 cm×2.1 mm id strong cation-exchange SCX column packed with 5 µm 300 Å polySULFOETHYL A-SCX material (PolyLC Inc., Columbia, MD) was used for fractionation. Fraction collection was done at three minute intervals using 30-min ammonium formate gradient (5–200 mM in 25% acetonitrile (ACN)) at 250 µL/min flow rate. From each biological replicate (*n*=3) nine peptide-containing fractions were chosen for liquid chromatography–tandem mass spectrometry (LC–MS/MS) analysis. A vacuum concentrator was used to dry peptides, which were resuspended in 10 µL of formic acid solution.

### 2.3 Liquid chromatography–tandem mass spectrometry (LC–MS/MS)

SCX peptides were applied to a 10 cm×75 µm id RP column (New Objective, Woburn, MA) packed with 5 µm 300 Å C18 material (ProteoPep II) and fractionated using a 180 minute linear gradient of 0.1% formic acid in ACN increased from 2% to 40%, increasing to 80% at 185 minutes through 190 min. A hybrid linear ion trap-Orbitrap instrument (LTQ Orbitrap XL, Thermo) was used to perform MS/MS analysis. With positive polarity in profile mode, a mass resolving power of 60,000, and a scan range of *m*/*z* 300–1650 Orbitrap full MS scans were collected. Further fragmentation in the ion trap was performed on the top ten most abundant ions. Global data dependent settings included dynamic exclusion duration of 300 s, with a repeat count of 30 s, and an exclusion list size of 500, and a repeat count of 1.

### 2.4 Database Searching

A quantitative proteomics software package for the analysis of large, high-resolution MS data sets, MaxQuant version 1.5.0.3, was used to analyze raw files. A current UniprotKB database containing *Mus musculus* (mouse) protein sequences and a second MaxQuant database of known contaminants were used to search the raw files with parameters including constant modification of cysteine by carbamidomethylation and variable modification of methionine oxidation. Additionally, multiplicity was set to 2, with a heavy set of lysine-6. The search tolerance and fragment ion mass tolerance were set to 6 ppm and 0.5 Da, respectively, at less than 1% false discovery rate. Perseus was used to perform statistical analysis, which evaluates the statistical significance of protein expression as described by Benjamini and Hochberg [4]. A threshold *q*-value of 0.05 was used for the Benjamini-Hochberg false discovery rate.
